# DEVELOPMENT OF PREDICTIVE MODELS FOR INCIDENT FRAILTY IN OLDER ADULTS: MACHINE LEARNING APPROACHES

**DOI:** 10.1093/geroni/igac059.3000

**Published:** 2022-12-20

**Authors:** Heeeun Jung, Miji Kim, Mina Park, Jinhyung Ryu, Kyung-Ryoul Mun, Chang Won Won

**Affiliations:** Kyung Hee University, Dongdaemun-gu, Seoul-t’ukpyolsi, Republic of Korea; Kyung Hee University, Seoul, Seoul-t’ukpyolsi, Republic of Korea; Korea University, Seongbuk-gu, Seoul-t’ukpyolsi, Republic of Korea; Korea Institute of Science and Technology, Seongbuk-gu, Seoul-t’ukpyolsi, Republic of Korea; Korea Institute of Science and Technology, Seongbuk-gu, Seoul-t’ukpyolsi, Republic of Korea; Kyung Hee University, Dongdaemun-gu, Seoul-t’ukpyolsi, Republic of Korea

## Abstract

Frailty is not only a clinical symptom accompanying adverse health outcomes but also a syndrome that involves a dynamic transition between varying phases. This study aimed to develop machine learning (ML) models for predicting incident frailty over a 2-year follow-up period among Korean community-dwelling adults aged 70 years and older. We conducted prospective analyses (n=2,408) among participants who underwent measurement of frailty status at baseline and 2-year follow-up from the Korean Frailty and Aging Cohort Study. Frailty was defined as the presence of three or more of five components using the Fried frailty phenotype. A total of 2,250 participants classified as non-frail at baseline and 116 (5.2%) with incident frailty during the 2-year follow-up. 32 variables selected by stepwise analysis were used as inputs among 77 variables composed of sociodemographic and clinical characteristics. The different ML models such as Logistic Regression(LR), K-Nearest Neighbor(KNN), Gaussian Naïve Base(NB), Support Vector Machine(SVM), and Random Forest(RF) with the 10-fold cross validation were compared. A feature selection, grid search, and data resampling methods were applied to find the best inputs and models. The best performing inputs of 16 variables were found and Gaussian NB achieved the highest accuracy of 0.82 and F1-score of 0.79 in predicting frailty incidence after 2-years with selected 16 inputs, followed by RF (accuracy 0.82, F1-score 0.76) and LR (accuracy 0.80, F1-score 0.76). We developed ML models for predicting frailty and is expected to contribute to the early prediction of frail older adults and timely intervention in clinical and community settings.

